# Supramolecular Weaving by Halogen-Bonding in Functionality-Rich Hexasubstituted Aromatic Synthons

**DOI:** 10.3390/ma16041678

**Published:** 2023-02-17

**Authors:** Matteo Catenazzi, Andrea Nitti, Massimo Boiocchi, Gabriele Bianchi, Riccardo Po, Dario Pasini

**Affiliations:** 1 Department of Chemistry and INSTM Research Unit, University of Pavia, Via Taramelli 12, 27100 Pavia, Italy; 2 Centro Grandi Strumenti, University of Pavia, Via Bassi 21, 27100 Pavia, Italy; 3 New Energies, Renewable Energies and Material Science Research Center, Eni SpA, Via Fauser 4, 28100 Novara, Italy

**Keywords:** hexasubstituted benzenes, halogen bond, supramolecular weaving, crystal engineering

## Abstract

Hexasubstituted benzenes are interesting platforms for the generation of functional materials, whose applications span from supramolecular recognition to organic electronics. Their synthesis is difficult to achieve by controlling multiple substitution steps of all hydrogen atoms on the aromatic benzene skeleton, so, often, cycloaddition reactions from disubsituted alkynes are used. In this work, we report a novel, straightforward route to *C*_3_-symmetrical hexasubstituted aromatic synthons with a diverse and rich pattern of functionalities, and we report about their packing mode in the crystals, in which, unprecedentedly, directional, strong halogen bonding interactions are capable of forming bidimensional supramolecular weaving.

## 1. Introduction

Benzene, the simplest aromatic organic structure, is one of the most common organic structures, and is widely used, impacting both technology and society. In fact, benzene is one of the most used building blocks in various functional organic materials, including pharmaceuticals, agrochemicals, plastics, and organic electronic devices. Despite its simplicity, the structural diversity that can be achieved by benzene derivatives is unexpectedly wide: benzene has six hydrogen atoms that can be replaced by a variety of substituents. For instance, according to Burnside’s counting theorem, the number of possible benzene molecules with six different substituents is 4291 [1]. Because of the lack of a general method to achieve multi-substituted benzenes and the inability to ensure regioselective protocols independent from the stereo-electronic characteristic of preinstalled substituents, the huge structural diversity allowed by substituted benzenes has not been fully exploited in chemistry [2].

**Figure 1:**
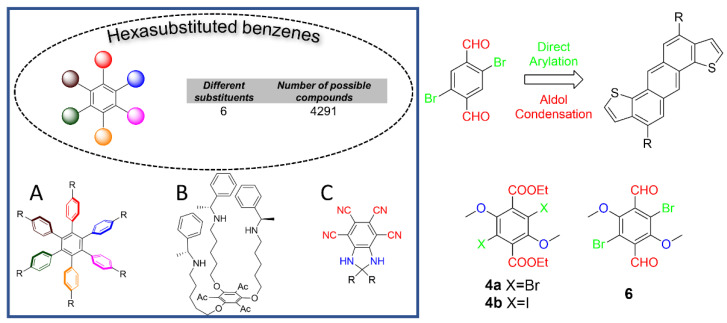
Schematic representation of hexa-substituted benzenes state-of-art. (**A**) hexaarylbenzenes; (**B**) *C*_3_-Symmetrical hexasubstituted benzenes; (**C**) Nonlinear optical materials built on hexasubstituted benzene moieties.

Hexasubstituted benzenes are an interesting platform for the generation of novel functional materials with tailored properties. For instance, hexaarylbenzenes (Figure 1A), whose synthesis is generally achieved via [4+2] cycloaddition or [2+2+2] co-trimeryzation of alkynes [3,4,5,6,7], have unique applications in materials science, including as liquid crystals [8,9], supramolecular electronic materials [10], organic electronics [11], molecular rotors [12], and redox materials [13], to cite a few. *C*_3_-Symmetrical hexasubstituted benzenes (Figure 1B) have been used as a platform for the construction of macrocycle systems in molecular recognition and supramolecular chemistry [14,15,16]. Nonlinear optical materials have been built on hexasubstituted benzene moieties (see Figure 1C) with a “push–pull” arrangements of the substituents [17,18]. Hexakis(phenylselenyl)benzene, a benzene ring with six phenylselenyl substituents, was studied as a platform to demonstrate the double aromaticity arising from σ- and π-conjugations [19].

We first became interested in hexasubstituted benzene substrates bearing an *o*-dibromodialdehyde substitution pattern, and with electron-donating alkoxy substituents in the remaining positions of the aromatic scaffold to complete the hexasubstitution of the parent benzene scaffold, such as molecular target **6**. In fact, our group developed a one-pot cascade process using *ortho*-bromoaldehyde substrates as starting materials, which involves direct arylation followed by an intramolecular cross-aldol condensation. This method has been proven to be a highly scalable and sustainable methodology for the annulation and rapid construction of conjugated compounds [20,21,22,23,24,25,26,27]. Some of these materials have been utilized for the construction of conjugated oligomers and polymers with outstanding sustainability and scalability characteristics for functional applications [28,29,30,31,32,33,34,35,36].

Crystal motifs of terephthalate esters (*p*-disubstituted aromatic esters) bearing two *ortho*-hydroxyl groups have been reported to show different polymorphic forms, depending on the twist angles of the ester groups with respect to the mean plane of the benzene ring [37,38,39,40]. The polymorphs are a consequence of the different motifs of intramolecular and intermolecular hydrogen bond (HB) interactions characterizing these compounds in the solid state.

In this work, we present the synthesis, characterization, and solid-state properties of novel hexasubstituted terephthalic esters **4** bearing halogen atoms and alkoxy groups. We demonstrate that our novel compounds pack through an ordered, bidimensional weaving of molecules in the solid state through specific halogen bonding (XB) interactions, to give millimeter-sized crystals, in which XB interactions are crucial for the packing [41,42,43]. Furthermore, we report a straightforward synthetic access to hexasubstituted dialdehyde **6**.

## 2. Results and Discussion

### 2.1. Synthesis

The known synthesis of **6** implies the oxidation of 1,4-dibromo-2,5-dimethoxy-3,6-dimethylbenzene, using the potassium salt of benzeneselenic acid as the oxidant [44]. This procedure was not reproducible because the starting material is not commercially available and its preparation has not been reported in a detailed manner.

**Scheme 1:**
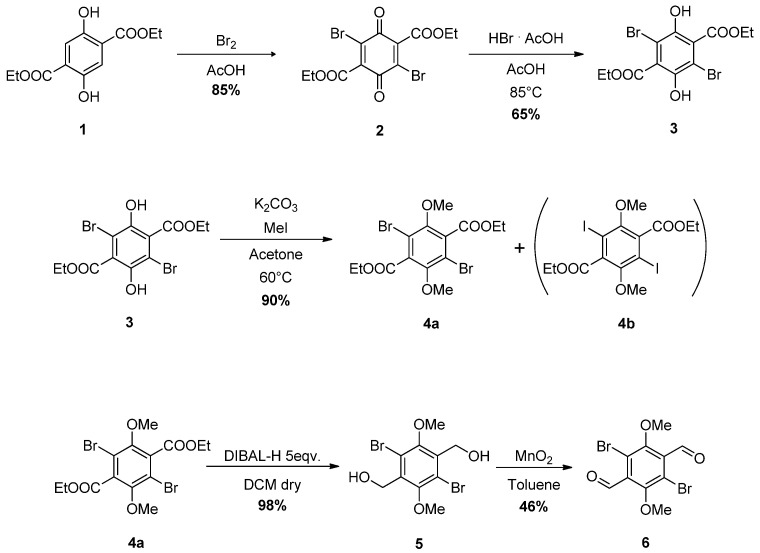
Synthesis of compounds **4a**,**b** and **6**.

Our synthetic approach is shown in Scheme 1 and consists of five synthetic steps using unexpensive reagents. The first two steps led to hexasubstituted benzene **3** according to our modification of a literature protocol that requires a bromination of compound **1**, to give tetrasubstituted-1,4-benzoquinone **2** in a high yield (85%), followed by its reduction with HBr (65% yield) [45]. Compound **3** was methylated using iodomethane in the presence of K_2_CO_3_ as a base in acetone, affording the new compound **4a** with 90% yield. Surprisingly, when this reaction was conducted in high dilution conditions (ca. 0.01 mol·L^−1^ instead of 0.3 mol·L^−1^) the formation of a new product **4b**, which was easy to separate from **4a** by flash-chromatography, was observed in a low yield. The low substrate concentration seemed to promote the partial substitution of bromine by the iodine atom of the methylating reagent, confirming the tendence of these compounds to react in nucleophilic aromatic substitution reactions [46]. In both the case of **4a** and **4b**, ^13^C NMR carbon resonances fall in the region of substituted benzenes, demonstrating that, even in the presence, such as in this case, of electron withdrawing and electron donating substituents, the aromaticity of the system is not substantially perturbed, with no quinoidal behavior associated to such compounds. NMR spectroscopy, single crystal XRD, and mass spectrometry analysis of compound **4b** confirm a structure with iodine atoms instead of bromine atoms. When trying to obtain **6**, the direct reduction with DIBAL starting from diester **4a** or **4b** failed, as well as our attempts to reduce **4a** or **4b** to dialcohol **5** with LiAlH_4_, as the aromatic dehalogenation took place concomitantly. Compound **4a** was then successfully reduced to alcohol **5** using an excess of DIBAL-H. Finally, compound **6** was oxidated with manganese dioxide to give dialdehyde **6**.

We immediately noticed that compound **4a** exhibited a strong tendency to form big and well-defined single crystals through the slow evaporation of a CH_2_Cl_2_ solution; we were also able to grow suitable single crystals from compound **4b**.

### 2.2. X-ray Crystal Structures

Single crystals 1 mm in length were obtained by slow evaporation of the DCM solutions of **4a** and **4b**. Both **4a** and **4b** compounds have an electrophilic region associated with the halogen atom (X) and a nucleophilic region associated to the lone pair of the oxygen atom of the carbonyl group (Y). Therefore, a typical R–X⋯Y halogen bonding interaction was expected to form, where R-X was the XB donor group and Y the XB acceptor [47,48,49].

**Figure 2:**
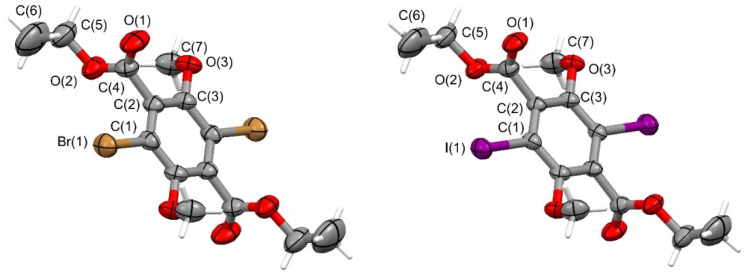
Thermal ellipsoids of the molecular compound **4a** (**left**) and **4b** (**right**) (ellipsoids are drawn at the 50% probability level; atom names are reported only for the asymmetric unit and an inversion center occurs at the center of the aromatic ring).

The crystallographic study shows that **4a** and **4b** compounds occurred as two isostructural crystals; plots showing the thermal ellipsoids are reported in Figure 2. The two molecular compounds had a perfect *C_i_* symmetry because an inversion center was placed at the center of the aromatic ring and the asymmetric unit was only a half of the molecular compound.

Both the ester groups and the methoxy arms were twisted out of the ring plane and placed accordingly in an antiperiplanar arrangement, with the two terminal ethyl chains, as well as the two terminal methyl arms, pointing towards opposite directions with respect to the plane of the aromatic ring, to impose a center of symmetry located at the center of the aromatic ring. The C atoms of the terminal ethyl groups were in the plane of the connected –COO– ester groups: the out of plane distances for the atoms involved were within 0.05(1) Å in **4a** and 0.03(1) Å in **4b**; the C(4)-O(2)-C(5)-C(6) torsion angle was 176.0(5)° in **4a** and 179.0(5) in **4b**. The ethyl-ester arms were almost perpendicular with respect to the plane of the aromatic ring, with a dihedral angle of 88.0(3)° in **4a** and 89.0(3)° in **4b**. Similarly, the methoxy groups were bent with respect to the best plane of the aromatic ring by 81.6(4)° in **4a** and 85.3(4)° in **4b**, as this resulted from the value of the dihedral angle between the best plane of the aromatic ring and the plane of the C(3)_Ar_-O(3)_methoxy_-C(7)_methoxy_ atoms. In both the molecular compounds, the C(1)′_Ar_-C(3)_Ar_-O(3)_methoxy_ bond angle (symmetry code: (′) = 1-*x*, -*y*, 1-*z*) increased from the ideal value of 120° to 122.2(3) in **4a** and 122.1(3)° in **4b**, and this widening suggests a repulsive interaction between the O_methoxy_ atom and the adjacent Br or I atoms.

On the contrary, supramolecular interactions occur between the halogen species and the oxygen of the carbonyl groups of the adjacent molecules. As both the bromine and the iodine molecular compounds have two halogen species and two carbonyl groups in the molecular moiety, each molecule is involved in four symmetrically equivalent C_Ar_-X⋯O_carbonyl_ halogen bonding interactions with four adjacent molecules; the C_Ar_-X group acts as the halogen bonding donor and the O_carbonyl_ atom acts as the halogen bonding acceptor. As for the bromide compound, the Br⋯O separation of 3.147(3) Å is shorter than the value of 3.37 Å, resulting from the sum of the van der Waals radii of bromine (1.85 Å) and oxygen (1.52 Å) [50]. The normalized *R* parameter (defined as the ratio between the observed X⋯Y separation and the sum of the proper radii of the involved species) is 0.93 and this value, together with an almost linear C_Ar_-Br⋯O_carbonyl_ angle of 172.1(1)°, confirm the presence of a well-established XB interaction.

As for the iodine compound, the I⋯O separation of 3.154(3) Å needed to be compared to a value of 3.50 Å, resulting from the sum of the iodine (1.98 Å) and oxygen radii [50]. The normalized *R* parameter decreased to 0.90 and suggests a stronger interaction, in agreement with the assessed fact that the strength of XB interactions is directly related to the polarizability of the XB donor atom, which follows the scale Br < I [47,48,49]. As a further probe of a stronger XB interaction in compound **4b**, the C-I⋯O angle in the iodine compound was more linear (174.7(1)°) than in the bromine counterpart.

Interestingly, in the iodine compound **4b**, an I⋯C contact of 3.653(4) Å, occurred between the halogen atom and the C(2) atom of the aromatic ring. This contact was a bit shorter than the value of 3.68 Å resulting from the sum of the van der Waals radii of iodine and carbon (1.70 Å) [50], thus suggesting that a weak halogen bond interaction occurred between the C_Ar_-I halogen bond donor group and the aromatic ring, which acted as a halogen bond acceptor. As stated above, the C_Ar_-Br group was less prone to creating strong halogen bond interactions, and in bromine compound **4a,** the Br⋯C contact involving the C(2) atom resulted in 3.660(3) Å, which was significantly longer than the value of 3.55 Å resulting from the sum of the van der Waals radii of the involved species.

**Figure 3:**
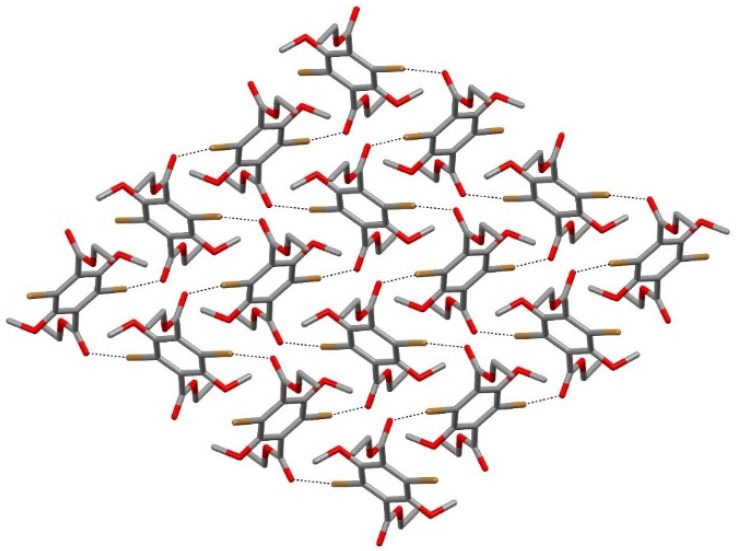
A simplified view of the halogen-bonded molecular sheet forming in the crystal of bromine compound **4a** (hydrogens were omitted for clarity; dashed lines indicate the C_Ar_-Br⋯O_carbonyl_ XB interactions). The same supramolecular arrangement occurs in the isostructural crystal of iodine compound **4b**.

In both compounds, the C_carbonyl_-O_carbonyl_⋯X angle of 112(1)° emphasized that the XB interaction involved the lone-pair of carbonyl oxygen in the nucleophilic (electron-rich) region. The supramolecular XB interactions originated at the solid state a bidimensional sheet of **4a** or **4b** molecules, extending parallel to the (100) plane. Figure 3 reports a simplified view of the halogen-bonded molecular sheet forming in the crystal of bromine compound **4a** and the same motif occurred in the crystal of iodine compound **4b**.

The comparison between the molecular structure of compound **4a** and the different polymorphic forms of the compound diethyl 2,5-dibromo-3,6-dihydroxyterephthalate [40], in which the same terephthalate ester carried two *ortho*-hydroxyl groups, allowed for establishing that the presence of an antiperiplanar arrangement for the ethyl-ester groups, coupled to a coplanar arrangement for atoms of the ethyl-ester arms, were a prerequisite for the establishment of XB interactions in the solid state.

**Figure 4:**
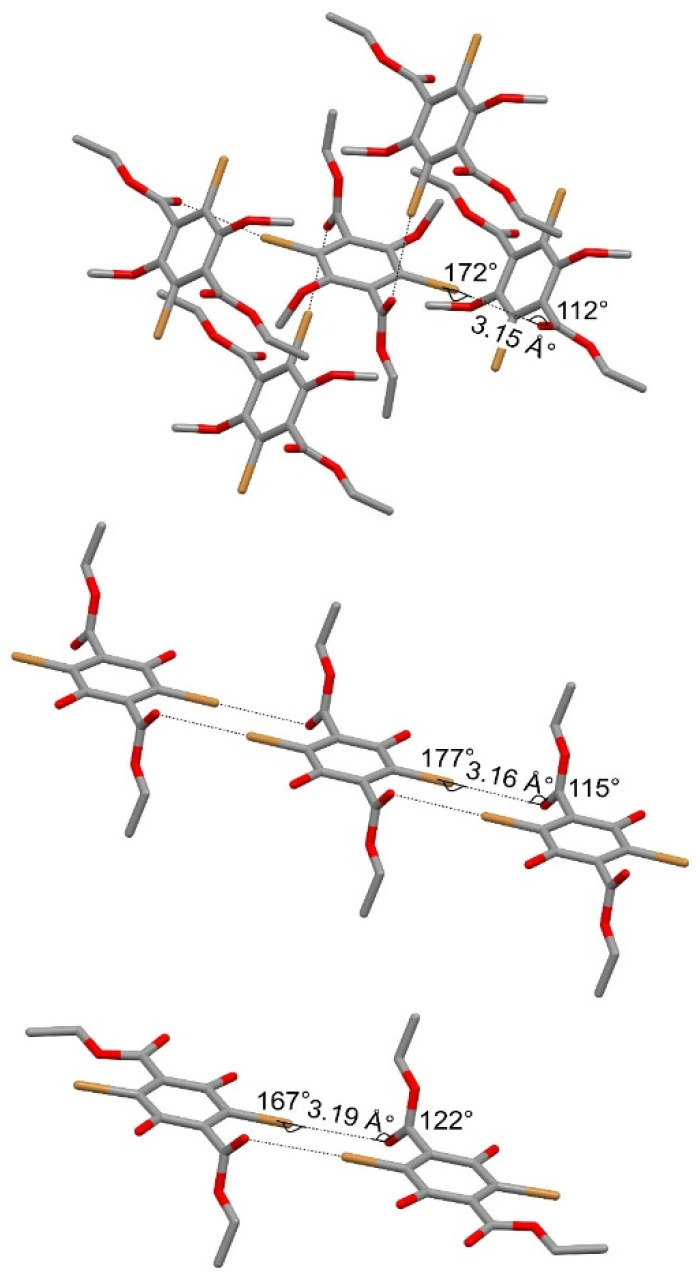
Simplified sketches of the supramolecular C_ar_-Br⋯O_carbonyl_ XB interactions (drawn as dashed lines) in the terephthalate-based compound **4a**, carrying two methoxy substituents on the aromatic ring (**top**), and in two polymorphic forms of diethyl 2,5-dibromo-3,6-dihydroxyterephthalate, carrying two hydroxy substituents: the form occurring in an ethanol solvate crystal (**middle**) and the form *II* (**bottom**). The XB motif on the top originates a supramolecular sheet, the one on the middle originates a supramolecular chain, the one on the bottom originates a supramolecular dimer.

As described above, in the **4a** compound, both ethyl-ester arms were arranged in such a manner and each molecule of **4a** was involved in four C_Ar_-Br⋯O_carbonyl_ XB interactions with four adjacent molecules (Figure 3 and Figure 4, top), leading to the formation of a supramolecular sheet.

The same conformation for the ethyl-ester groups occurred in the form of compound diethyl 2,5-dibromo-3,6-dihydroxyterephthalate occurring in an ethanol solvate crystal [40], and in this case, each molecule of diethyl 2,5-dibromo-3,6-dihydroxyterephthalate was involved in four C_Ar_-Br⋯O_carbonyl_ XB interactions. However, in the ethanol solvate crystal of diethyl 2,5-dibromo-3,6-dihydroxyterephthalate, each molecular compound originated couples of XB interactions with two adjacent molecules (Figure 4 middle), leading to the formation of a supramolecular chain.

On the contrary, in the form *II* of diethyl 2,5-dibromo-3,6-dihydroxyterephthalate [40], only one of the ethyl-ester arms was twisted out of the aromatic plane and only two C_Ar_-Br⋯O_carbonyl_ XB interactions occurred. These interactions (Figure 4 bottom) involved the bromide atom near the twisted ethyl-ester arm and the carbonyl oxygen of the twisted ethyl-ester arm of an adjacent molecule, creating a supramolecular dimer. As shown by the geometrical features reported in Figure 4, in all of the structures, similar XB interactions with normalized *R* parameters ranging from 0.90 to 0.95 occurred.

Interestingly, the form *I* of diethyl 2,5-dibromo-3,6-dihydroxyterephthalate [40] exhibited an antiperiplanar arrangement of the two ester groups, but both the terminal ethyl chains were folded out of the plane of the COO ester atoms and in the solid state, XB interactions did not occur for such molecular conformations. Clearly, in this form of diethyl 2,5-dibromo-3,6-dihydroxyterephthalate, as well as in the other two forms described above, additional HB interactions originated, with the OH group of the hydroxy substituent able to act as a proton-donor group.

## 3. Conclusions

We developed a novel synthetic route for the synthesis of a functionality-rich, *C*_3_-symmetrical hexasubstituted benzenes **4** and **6**. The synthesis of dialdehyde **6**, while involving five synthetic steps, was simple to implement, as the reaction conditions were always mild, and the intermediate products were obtained without the need to resort to purification by chromatography. Furthermore, we demonstrated, with a thorough crystallographic study, the possibility of both derivatives **4a** and **4b** to form a well defined, two-dimensional supramolecular weaving in the crystal. The introduction of protected methoxy functionalities, instead of the unprotected hydroxy functionalities reported in the literature, activated the unicity of XB interactions, and provided very useful data for the construction of elaborated synthons for crystal engineering and, eventually, metal organic frameworks. The applicability of molecule **6** in the framework of scalable synthetic annulation strategies, for the expansion of the π-scaffold, and their integration into functional π-conjugated oligomers and polymers is currently under investigation in our laboratories.

## 4. Experimental Part

All commercially available reagents and solvents were used as received. The starting material and reagents were bought from Sigma Aldrich, TCI, Alfa Aesar, and Fluorochem. Analytical thin layer chromatography (TLC) was performed on chromophore loaded, commercially available Silica gel 60 F254 plates (Merck). Flash chromatography was carried out using Merck silica gel (pore size 60 Å, 230–400 mesh). ^1^H and ^13^C NMR spectra were recorded from solutions in CDCl_3_ on AC-200 or AMX-300 Bruker spectrometers using the solvent residual proton signal or tetramethylsilane (TMS) as the internal standard. Chemical shifts were expressed in ppm downfield from TMS in δ units. Samples for the mass spectrometry were analyzed with GC-MS and ESI-MS Agilent conventional instrumentation. Diffraction data for **4a** and **4b** were collected by means of an Enraf-Nonius CAD4 conventional diffractometer (Enraf-Nonius, Delft, The Netherlands), working at ambient temperature with graphite-monochromatized Mo Kα X-radiation (λ = 0.7107 Å). Data reductions were performed with the WinGX package [51]; the absorption effects were evaluated with the ψ-scan method [52], and absorption correction was applied to the data. All crystal structures were solved using direct methods (SIR 97) [53] and refined by full-matrix least-squares procedures on *F*^2^ using all reflections (SHELXL 2018/3) [54]. Anisotropic displacement parameters were refined for all non-hydrogen atoms; hydrogens were placed at calculated positions with the appropriate AFIX instructions and refined using a riding model. Crystal data for the studied molecular compounds are reported in Table S1. CCDC 2235890 and 2235891 contained the supplementary crystallographic data for this paper. These data can be obtained free of charge from the Cambridge Crystallo-graphic Data Centre.

*Preparation of compound***2**. A solution of Br_2_ (6.7 mL, 130 mmol) in AcOH (60 mL) was added dropwise to a solution of diethyl 2,5-dihydroxyterephthalate (3.3 g, 13 mmol) in AcOH (70 mL). H_2_O (40 mL) was finally added. The resulting mixture remained under magnetic stirring for 24 h, and was then filtered to give diethyl 2,5-dibromo-3,6-dioxocyclohexa-1,4-diene-1,4-dicarboxylate (4.56 g, 86%) as a yellow powder. ^1^H NMR (200 MHz, CDCl_3_) *δ*: 4.45 (q, *J* = 7.1 Hz, 4H), 1.39 (t, *J* = 7.1 Hz, 6H).

*Preparation of compound***3**. A 33% *w*/*w* solution of HBr in AcOH (1.39 mL) was added dropwise at 85 °C, under magnetic stirring, to a suspension of compound **2** (815 mg, 2 mmol) in AcOH (1.5 mL). The Br_2_ vapors that developed were occasionally removed by means of an air stream. After 2 h, the reaction mixture was extracted with Et_2_O. The organic phase was washed with H_2_O, sat. aq. NaHCO_3_ and H_2_O, dried on Na_2_SO_4_, filtered, and concentrated under reduced pressure to give the crude product, which was recrystallized in CH_2_Cl_2_/hexane. Filtration and washing of the solid with a little hexane gave the pure product diethyl 2,5-dibromo-3,6-dihydroxyterephthalate (**3**) as a white solid (528 mg, 64%). ^1^H NMR (300 MHz, CDCl_3_) *δ*: 8.97 (s, 2H), 4.50 (q, *J* = 7.1 Hz, 4H), 1.45 (t, *J* = 7.1 Hz, 6H).

*Preparation of compound***4a**. MeI (4.69 mL, 74.67 mmol) was added to a solution of compound **3** (3.08 g, 7.47 mmol) and K_2_CO_3_ (6.19 g, 44.8 mmol) in acetone (25 mL). The resulting mixture was kept under stirring at 60 °C for 24 h, then the solvent was removed under reduced pressure. The obtained solid was dissolved in DCM. The organic phase was washed with H_2_O and brine, dried on Na_2_SO_4_, filtered, and concentrated under reduced pressure to give the crude product, which was filtered through silica gel to give the pure product diethyl 2,5-dibromo-3,6-dimethoxyterephthalate as a yellow crystalline solid (2.95 g, 90%). ^1^H NMR (300 MHz, CDCl_3_) *δ*: 4.45 (q, *J* = 7.1 Hz, 4H), 3.88 (s, 6H), 1.41 (t, *J* = 7.1 Hz, 6H). ^13^C NMR (75 MHz, CDCl_3_) *δ*: 164.27, 150.94, 134.02, 113.78, 62.58, 62.39, 13.99. *Preparation of compound* **4b**. MeI (0.23 mL, 3.7 mmol) was added to a solution of compound **3** (0.15 g, 0.37 mmol) and K_2_CO_3_ (0.31 g, 2.24 mmol) in acetone (30 mL). The resulting mixture was kept under stirring at 60 °C for 24 h, then the solvent was removed under reduced pressure. The obtained solid was dissolved in DCM. The organic phase was washed with H_2_O and brine, dried on Na_2_SO_4_, filtered, and concentrated under reduced pressure to give the crude product, which was filtered through silica gel to give both **4a** and **4b** (80% overall yield) in a 2:1 ratio. The NMR spectra of the two products were superimposable.

*Preparation of compound* **5**. A 1M solution of DIBAL-H in hexane (6.8 mL, 6.8 mmol) was added dropwise, at −78 °C, to a solution of compound **4a** (600 mg, 1.36 mmol) in anhydrous DCM (13 mL). After 1 h at −78 °C, the reaction mixture remained under stirring at room temperature for 3 days, and was then quenched with HCl conc., stirred for 1 h, and finally diluted with H_2_O and extracted with DCM. The organic phase was dried on Na_2_SO_4_, filtered, and concentrated under reduced pressure to give the pure product (2,5-dibromo-3,6-dimethoxy-1,4-phenylene)dimethanol **5** as a white solid (472 mg, 98%). ^1^H NMR (300 MHz, CDCl_3_) *δ*: 4.88 (s, 4H), 3.91 (s, 6H), 1.85 (s, 2H). ^13^C NMR (75 MHz, CDCl_3_) *δ*: 153.14, 135.55, 119.82, 62.43, 60.64. GC-MS (ESI) *m/z*: 355 [M + H]^+^.

*Preparation of compound* **6**. MnO_2_ (1.10 g, 12.6 mmol) was added to a solution of compound **5** (450 mg, 1.26 mmol) in toluene (20 mL). The reaction mixture remained under stirring, at 100 °C, for 24 h, and was then filtered on celite and evaporated under reduced pressure to give the crude product. The latter was purified by a short silica gel pad (eluent Hexane:AcOEt 8:2) to give the pure product 2,5-dibromo-3,6-dimethoxyterephthalaldehyde as a yellow crystalline solid (203 mg, 46%). ^1^H NMR (200 MHz, CDCl_3_) *δ*: 10.27 (s, 2H), 3.94 (s, 6H). ^13^C NMR (75 MHz, CDCl_3_) *δ*: 189.14, 156.23, 133.65, 119.48, 63.53.

## Data Availability

The data presented in this study are available in the Supplementary Materials section.

## Supplementary Materials

The following supporting information can be downloaded at: https://www.mdpi.com/article/10.3390/ma16041678/s1. Table S1: crystal data for investigated crystals. Figures S1–S9: copies of NMR and GC spectra of the compounds.

## References

[1] Polya G., Reade R.C. (1987). Combinatorial Enumerations of Groups, Graphs, and Chemical Compounds.

[2] Itami K., Yoshida J. (2006). Platform synthesis: A useful strategy for rapid and systematic generation of molecular diversity. Chem. Eur. J..

[3] Suzuki S., Segawa Y., Itami K., Yamaguchi J. (2015). Synthesis and characterization of hexaarylbenzenes with five or six different substituents enabled by programmed synthesis. Nat. Chem..

[4] Hoye T.R., Baire B., Niu D., Willoughby P.H., Woods B.P. (2012). The hexadehydro-Diels–Alder reaction. Nature.

[5] Domínguez G., Pérez-Castells J. (2011). Recent advances in [2+2+2] cycloaddition reactions. Chem. Soc. Rev..

[6] Geng Y., Fechtenkötter A., Müllen K. (2001). Star-like substituted hexaarylbenzenes: Synthesis and mesomorphic properties. J. Mater. Chem..

[7] Chinchilla R., Najera C. (2014). Chemicals from alkynes with palladium catalysts. Chem. Rev..

[8] Nagata T., Obora Y. (2020). Transition-Metal-Mediated/Catalyzed Synthesis of Pyridines, Pyrimidines, and Triazines by [2+2+2]Cycloaddition Reactions. Asian J. Org. Chem..

[9] Short R., Carta M., Bezzu C.G., Fritsch D., Kariuki B.M., McKeown N.B. (2011). Hexaphenylbenzene-based polymers of intrinsic microporosity. Chem. Commun..

[10] Tomović Z., van Dongen J., George S.J., Xu H., Pisula W., Leclère P., Smulders M.M.J., De Feyter S., Meijer E.W., Schenning A.P.H.J. (2007). Star-shaped oligo(p-phenylenevinylene) substituted hexaarylbenzene: Purity, stability, and chiral self-assembly. J. Am. Chem. Soc..

[11] Devibala P., Balambiga B., Noureen S., Nagarajan S. (2021). Hexaarylbenzene based high-performance p-channel molecules for electronic applications. RSC Adv..

[12] Hiraoka S., Hisanaga Y., Shiro M., Shionoya M. (2010). A molecular double ball bearing an AgI–PtII dodecanuclear quadruple-decker complex with three rotors. Angew. Chem. Int. Ed..

[13] Steeger M., Lambert C. (2012). Charge-transfer interactions in tris-donor–tris-acceptor hexaarylbenzene redox chromophores. Chem. Eur. J..

[14] Choksakulporn S., Punkvang A., Sritana-anant Y. (2015). Synthesis and amino acids complexation of tripodal hexasubstituted benzene chiral receptors. J. Mol. Struct..

[15] Carrillo R., Martín T., López-Rodríguez M., Pinacho Crisóstomo F. (2014). Expedient Synthesis of C3-Symmetric Hexasubstituted Benzenes via Nicholas Reaction/[2+2+2] Cycloaddition. New Platforms for Molecular Recognition. Org. Lett..

[16] Wei J., Gao Y., Ma X., Jia X., Shia X., Chen Z. (2010). A novel class of C3d symmetrical molecules synthesized by a six-fold substitution from 1,4,5,8,9,12-hexabromododecahydrotriphenylene. Chem. Commun..

[17] Traber B. (2004). Hexasubstituted donor–acceptor benzenes as nonlinear optically active molecules with multiple charge-transfer transitions. Chem. Eur. J..

[18] Wudarczyk J., Papamokos G., Margaritis V., Schollmeyer D., Hinkel F., Baumgarten M., Floudas G., Müllen K. (2016). Hexasubstituted Benzenes with Ultrastrong Dipole Moments. Angew. Chem. Int. Ed..

[19] Furukawa S., Fujita M., Kanatomi Y., Minoura M., Hatanaka M., Morokuma K., Ishimura K., Saito M. (2018). Double aromaticity arising from σ- and π-rings. Chem. Commun..

[20] Nitti A., Bianchi G., Po R., Swager T.M., Pasini D. (2017). Domino Direct Arylation and Cross-Aldol for Rapid Construction of Extended Polycyclic π-Scaffolds. J. Am. Chem. Soc..

[21] Nitti A., Bianchi G., Po R., Pasini D. (2019). Scalable Synthesis of Naphtothiophene and Benzodithiophene Heterocyclic Scaffolds for Organic Electronics. Synthesis.

[22] Nitti A., Osw P., Calcagno G., Botta C., Etkind S.I., Bianchi G., Po R., Swager T.M., Pasini D. (2020). One-Pot Regiodirected Annulations for the Rapid Synthesis of π-Extended Oligomers. Org. Lett..

[23] Nitti A., Bianchi G., Po R., Porta A., Galbiati A., Pasini D. (2019). Weiss-Cook Condensations for the Synthesis of Bridged Bithiophene Monomers and Polymers. ChemistrySelect.

[24] Nitti A., Osw P., Abdullah M.N., Galbiati A., Pasini D. (2018). Scalable Synthesis of Naphthothiophene-based D-π-D Extended Oligomers through Cascade Direct Arylation Processes. Synlett.

[25] Bianchi G., Carbonera C., Ciammaruchi L., Camaioni N., Negarville N., Tinti F., Forti G., Nitti A., Pasini D., Facchetti A. (2022). An Anthradithiophene Donor Polymer for Organic Solar Cells with a Good Balance between Efficiency and Synthetic Accessibility. Sol. RRL.

[26] Forti G., Nitti A., Bianchi G., Po R., Pasini D. (2022). A Sustainable Synthetic Approach to the Indaceno [1,2-b:5,6-b′]dithiophene (IDT) Core through Cascade Cyclization–Deprotection Reactions. Chemistry.

[27] Nitti A., Forti G., Bianchi G., Botta C., Tinti F., Gazzano M., Camaioni N., Po R., Pasini D. (2021). Anthradithiophene-based organic semiconductors through regiodirected double annulations. J. Mater. Chem. C.

[28] Coluccini C., Dondi D., Caricato M., Taglietti A., Boiocchi M., Pasini D. (2010). Structurally-variable, rigid and optically-active D2 and D 3 macrocycles possessing recognition properties towards C 60. Org. Biomol. Chem..

[29] Colombo S., Coluccini C., Caricato M., Gargiulli C., Gattuso G., Pasini D. (2010). Shape selectivity in the synthesis of chiral macrocyclic amides. Tetrahedron.

[30] Penconi M., Bianchi G., Nitti A., Savoini A., Carbonera C., Pasini D., Po R., Luzzati S. (2021). A Donor Polymer with a Good Compromise between Efficiency and Sustainability for Organic Solar Cells. Adv. Energy Sustain. Res..

[31] Corsini F., Nitti A., Tatsi E., Mattioli G., Botta C., Pasini D., Griffini G. (2021). Large-Area Semi-Transparent Luminescent Solar Concentrators Based on Large Stokes Shift Aggregation-Induced Fluorinated Emitters Obtained Through a Sustainable Synthetic Approach. Adv. Opt. Mater..

[32] Asakawa M., Brown C.L., Pasini D., Stoddart J.F., Wyatt P.G. (1996). Enantioselective recognition of amino acids by axially-chiral π-electron-deficient receptors. J. Org. Chem..

[33] Osw P., Nitti A., Abdullah M.N., Etkind S.I., Mwaura J., Galbiati A., Pasini D. (2020). Synthesis and Evaluation of Scalable D-A-D π-Extended Oligomers as p-Type Organic Materials for Bulk-Heterojunction Solar Cells. Polymers.

[34] Havard J.M., Yoshida M., Pasini D., Vladimirov N., Frechet J.M.J., Medeiros D.R., Patterson K., Yamada S., Willson C.G., Byers J.D. (1999). Design of Photoresists with Reduced Environmental Impact. 2. Water-soluble Resists Based on Photocrosslinking of Poly(2-isopropenyl-2-oxazoline). J. Polym. Sci. A Polym. Chem..

[35] Virgili T., Forni A., Cariati E., Pasini D., Botta C. (2013). Direct evidence of torsional motion in an aggregation-induced emissive chromophore. J. Phys. Chem. C.

[36] Klopp J.M., Pasini D., Frechet J.M.J., Willson C.G., Byers J.D. (2001). Microlithographic Assessment of a Novel Family of Transparent and Etch Resistant Chemically Amplified 193 nm Resists Based on Cyclopolymers. Chem. Mater..

[37] Hantzsch A. (2015). The chromoisomerism of p-dihydroxyterephthalic acid derivatives as phenol-enol isomerism. Berichte Dtsch. Chem. Ges..

[38] Byrn S.R., Curtin D.Y., Paul I.C. (1972). The X-ray Crystal Structures of the Yellow and White Forms of Dimethyl 3,6-Dichloro-2,5-dihydroxyterephthalate and a Study of the Conversion of the Yellow Form to the White Form in the Solid State. J. Am. Chem. Soc..

[39] Yang Q.C., Richardson M.F., Dunitz J.D. (1985). Internal Molecular Motion as Forerunner of a Phase Change Involving Conformational Isomerization. J. Am. Chem. Soc..

[40] Näther C., Nagel N., Bock H., Seitz W., Havlas Z. (1996). Structural, kinetic and thermodynamic aspects of the conformational dimorphism of di ethyl 3,6-di bromo-2,5-di hydroxyterephthalate. Acta Crystallogr. B.

[41] Benniston A.C., Winstanley T.P.L., Lemmetyinen H., Tkachenko N.V., Harrington R.W., Wills C. (2012). Large Stokes Shift Fluorescent Dyes Based on a Highly Substituted Terephthalic Acid Core. Org. Lett..

[42] Nitti A., Botta C., Forni A., Cariati E., Lucenti E., Pasini D. (2020). Crystallization-induced room-temperature phosphorescence in fumaramides. CrystEngComm.

[43] Botta C., Benedini S., Carlucci L., Forni A., Marinotto D., Nitti A., Pasini D., Righetto S., Cariati E. (2016). Polymorphism-dependent aggregation induced emission of a push–pull dye and its multi- stimuli responsive behavior. J. Mater. Chem. C.

[44] Syper L., Młochowski J. (1984). A convenient oxidation of halomethylarenes and alcohols to aldehydes with dimethyl selenoxide and potassium benzeneselenite. Synthesis.

[45] Hintermann L., Altmann P.J., Naumov P., Suzuki K. (2017). Synthesis of Tetraoxygenated Terephthalates via a Dichloroquinone Route: Characterization of Cross-Conjugated Liebermann Betaine Intermediates. Helv. Chim. Acta.

[46] Hintermann L., Masuo R., Suzuki K. (2008). Solvent-Controlled Leaving-Group Selectivity in Aromatic Nucleophilic Substitution. Org. Lett..

[47] Bolton O., Lee K., Kim H.-J., Lin K.Y., Kim J. (2011). Activating efficient phosphorescence from purely organic materials by crystal design. Nat. Chem..

[48] Metrangolo P., Neukirch H., Pilati T., Resnati G. (2005). Halogen Bonding Based Recognition Processes:  A World Parallel to Hydrogen Bonding. Acc. Chem. Res..

[49] Fox D., Metrangolo P., Pasini D., Pilati T., Resnati G. (2008). Terraneo, Site Selective Supramolecular Synthesis of Halogen Bonded Cocrystals Incorporating the Photoactive Azo Group. CrystEngComm.

[50] Bondi A. (1964). Van der Waals Volumes and Radii. J. Phys. Chem..

[51] Farrugia L.J. (2012). WinGX and ORTEP for Windows: An update. J. Appl. Crystallogr..

[52] North A.C.T., Phillips D.C., Mathews F.S. (1968). A Semi-Empirical Method of Absorption Correction. Acta Crystallogr. Sect. A.

[53] Altomare A., Burla M.C., Camalli M., Cascarano G.L., Giacovazzo C., Guagliardi A., Moliterni A.G.G., Polidori G., Spagna R. (1999). SIR97: A new tool for crystal structure determination and refinement. J. Appl. Crystal..

[54] Sheldrick G.M. (2015). Crystal Structure Refinement with SHELXL. Acta Crystallogr. Sect. C.

