# Supplementary material for: Supramolecular Weaving by Halogen-Bonding in Functionality-Rich Hexasubstituted Aromatic Synthons
Source: Materials (Basel). 2023 Feb 17;16(4):1678. doi: 10.3390/ma16041678 (PMC9967865; doi:10.3390/ma16041678)

Supporting Information for:

Supramolecular Weaving by Halogen-Bonding in Functionality-Rich Hexasubstituted Aromatic Synthons

Matteo Catenazzi,[1] Andrea Nitti,[1] Massimo Boiocchi,[2] Gabriele Bianchi,[3] Riccardo Po,[3] and Dario Pasini[1,*]

1 Department of Chemistry and INSTM research unit, University of Pavia, Via Taramelli 12, 27100 Pavia, Italy

2 Centro Grandi Strumenti, University of Pavia, Via Bassi 21, 27100 Pavia, Italy

3 New Energies, Renewable Energies and Material Science Research Center, Eni SpA, Via Fauser 4, 28100 Novara, Italy

* Correspondence: dario.pasini@unipv.it

Table S1 Crystal data for investigated crystals.

	**4a**	**4b**
Formula	$C_{14}H_{16}Br_2O_6$	$C_{14}H_{16}I_2O_6$
M	440.07	534.07
Dimension [mm]	0.58 x 0.43 x 0.32	0.58 x 0.30 x 0.14
Colour	colourless	colourless
Crystal system	monoclinic	monoclinc
Space group	$P2_1/c$ (no. 14)	$P2_1/c$ (no. 14)
a [Å]	11.241(3)	11.076(2)
b [Å]	9.3984(9)	9.4609(9)
c [Å]	8.3297(14)	8.7093(11)
β [°]	99.335(12)	100.318(7)
V [Å^3]	868.4(5)	897.9(2)
Z	2	2
ρ_{calcd} [g cm^{-3}]	1.683	1.975
μ Mo$_{K\alpha}$ [mm^{-1}]	4.690	3.525
Scan type	ω scans	ω scans
θ range [°]	2.8–28.3	2.8–30.0
Measured reflections	3164	5103
Unique reflections	2156	2618
R_{int}	0.018	0.034
Strong data [$I_O > 2\sigma(I_O)$]	1859	2035
Refined parameters	103	102
R1, wR2 (strong data)	0.0438, 0.0537	0.0379, 0.0513
R1, wR2 (all data)	0.0965, 0.1063	0.0925, 0.1037
GOF	1.248	1.038
Max/min residuals [eÅ^{-3}]	0.43/–0.26	0.72/–0.66

Copie of NMR spectra

Diethyl 2,5-dibromo-3,6-dioxocyclohexa-1,4-diene-1,4-dicarboxylate (2)

Figure S1. ^{1}H NMR (200 MHz, $CDCl_3$)

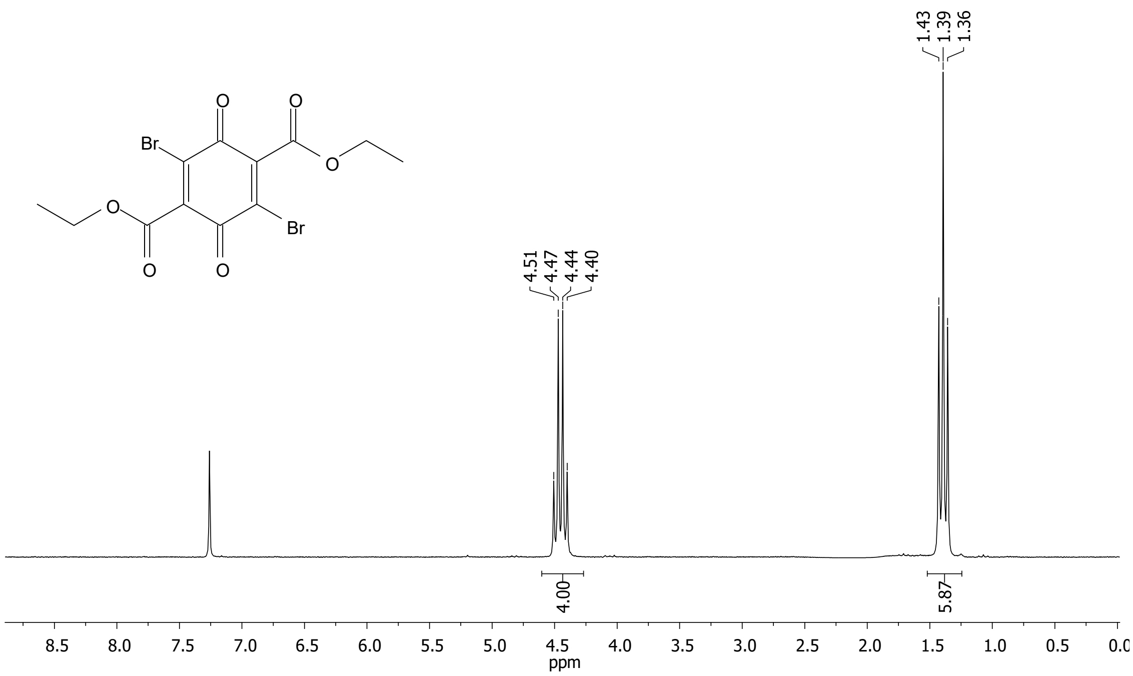

Diethyl 2,5-dibromo-3,6-dihydroxyterephthalate (3)

Figure S2. ^{1}H NMR (200 MHz, $CDCl_3$)

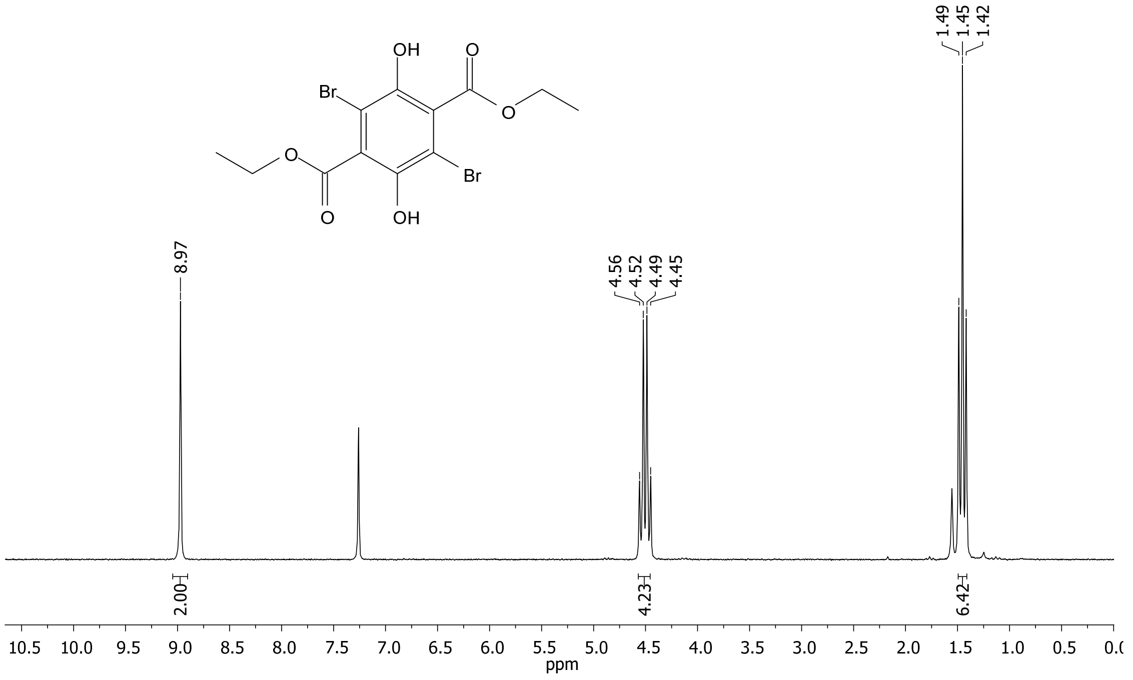

Diethyl 2,5-dibromo-3,6-dimethoxyterephthalate (4a)

Figure S3. ^{1}H NMR (200 MHz, $CDCl_3$)

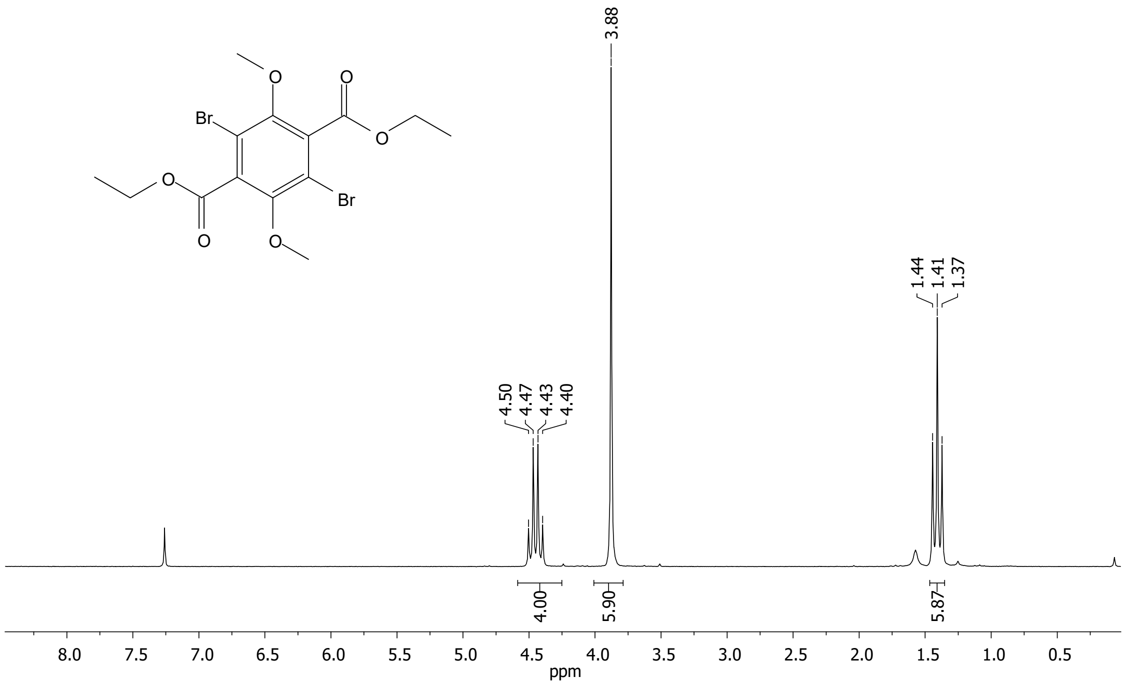

Figure S4. ^{13}C NMR (75 MHz, $CDCl_3$)

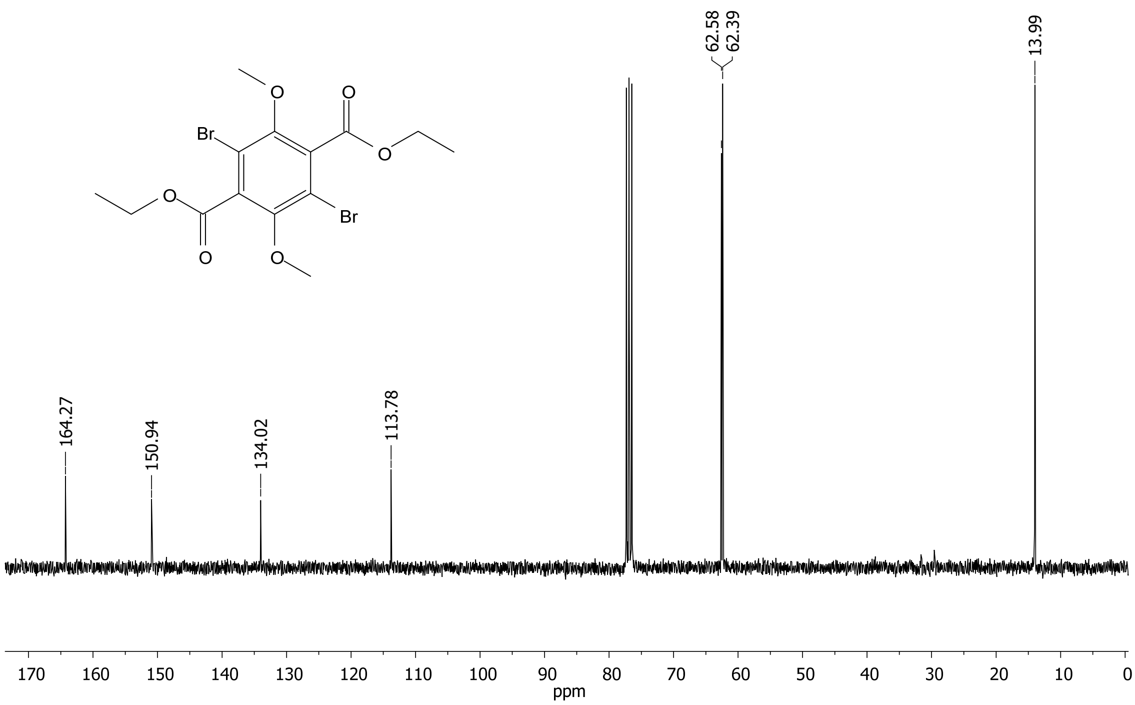

2,5-Dibromo-3,6-dimethoxy-1,4-phenylenedimethanol (5)

Figure S5. ^{1}H NMR (200 MHz, $CDCl_3$)

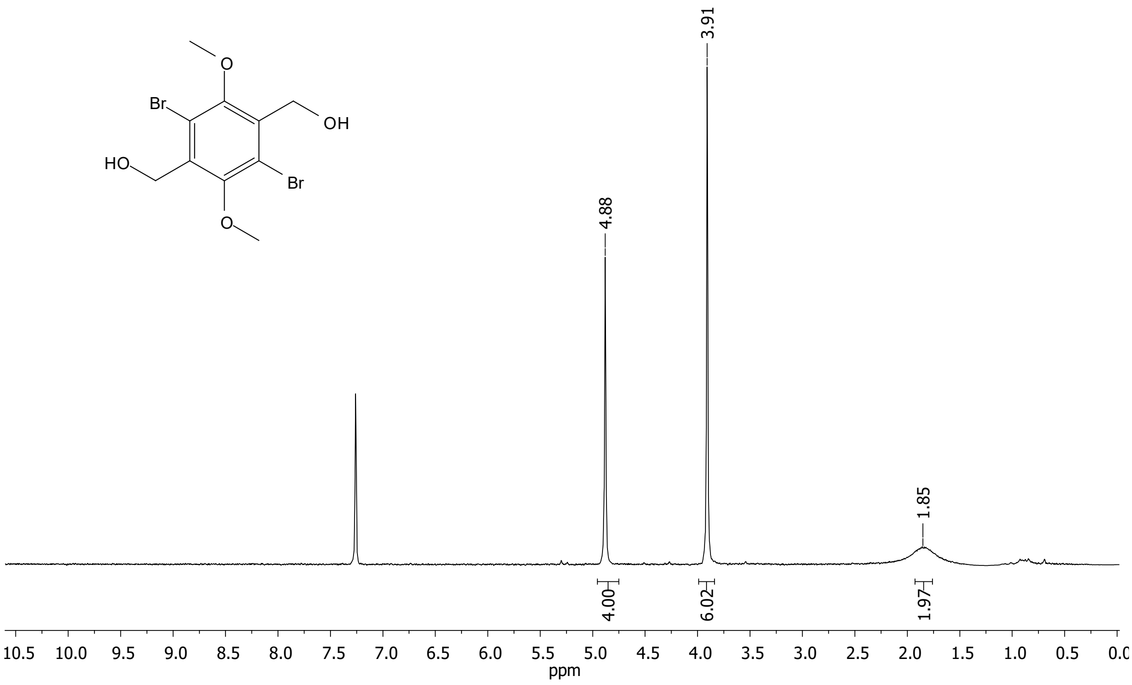

Figure S6. ^{13}C NMR (75 MHz, $CDCl_3$)

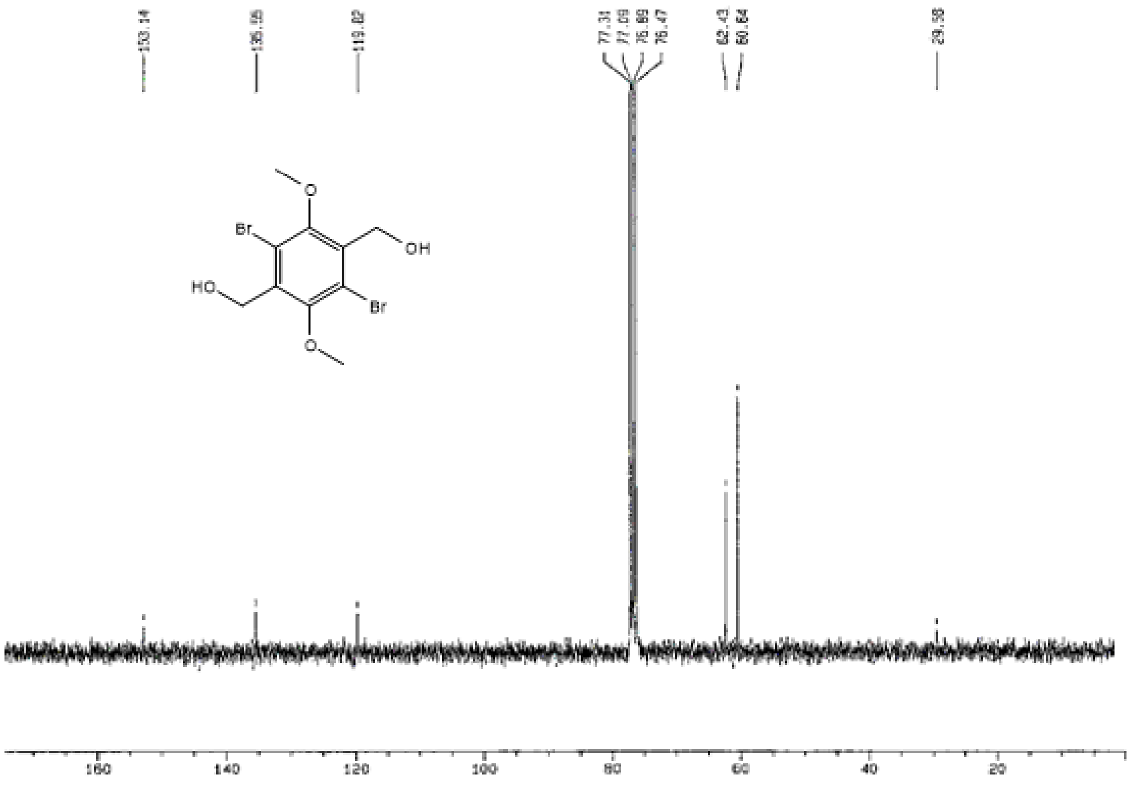

Figure S7. GC-MS

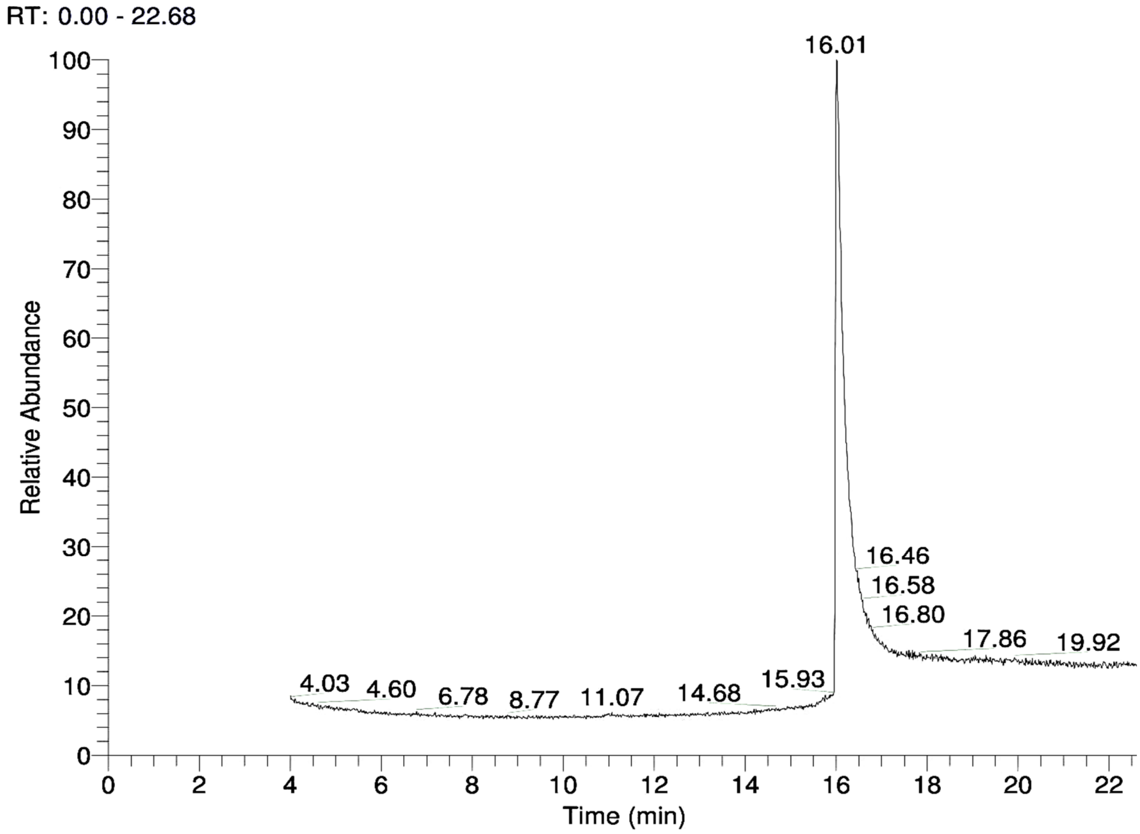

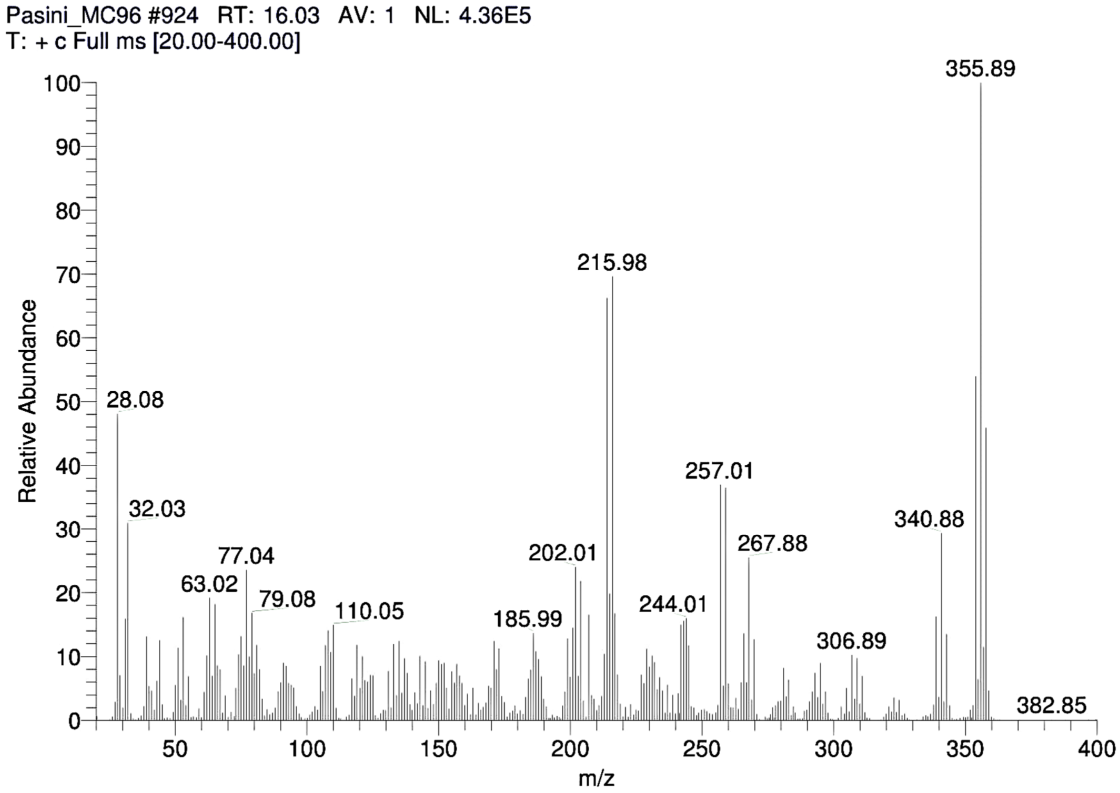

2,5-dibromo-3,6-dimethoxyterephthalaldehyde (6)

Figure S8. ^{1}H NMR (200 MHz, $CDCl_3$)

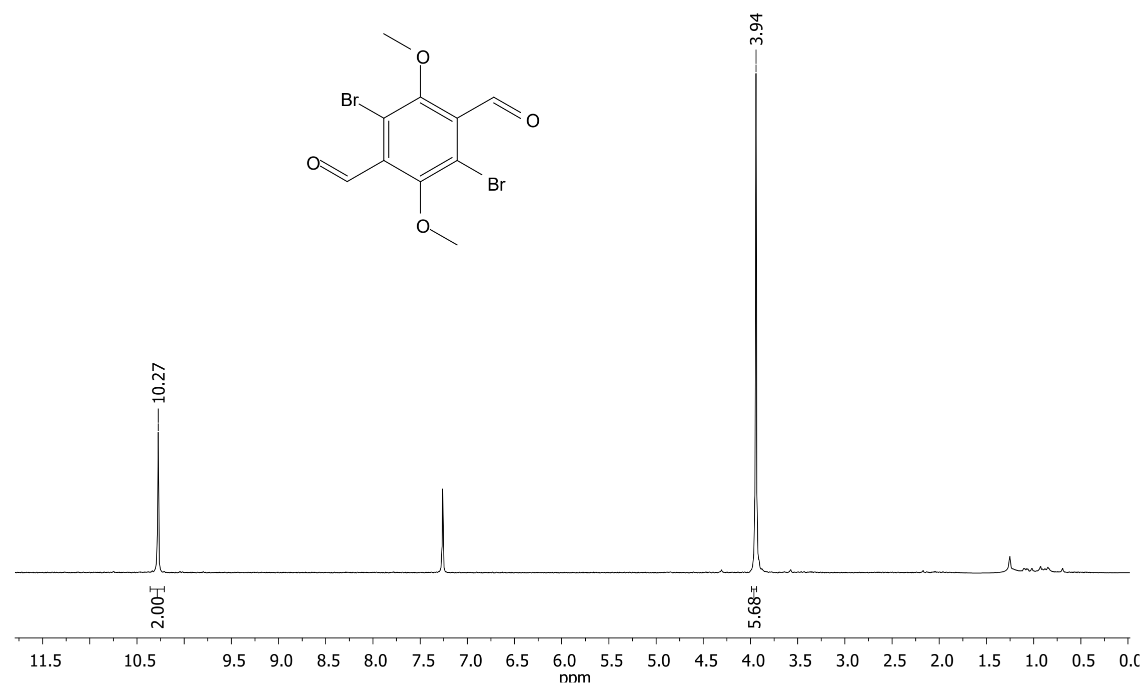

Figure S9. ^{13}C NMR (75 MHz, $CDCl_3$)

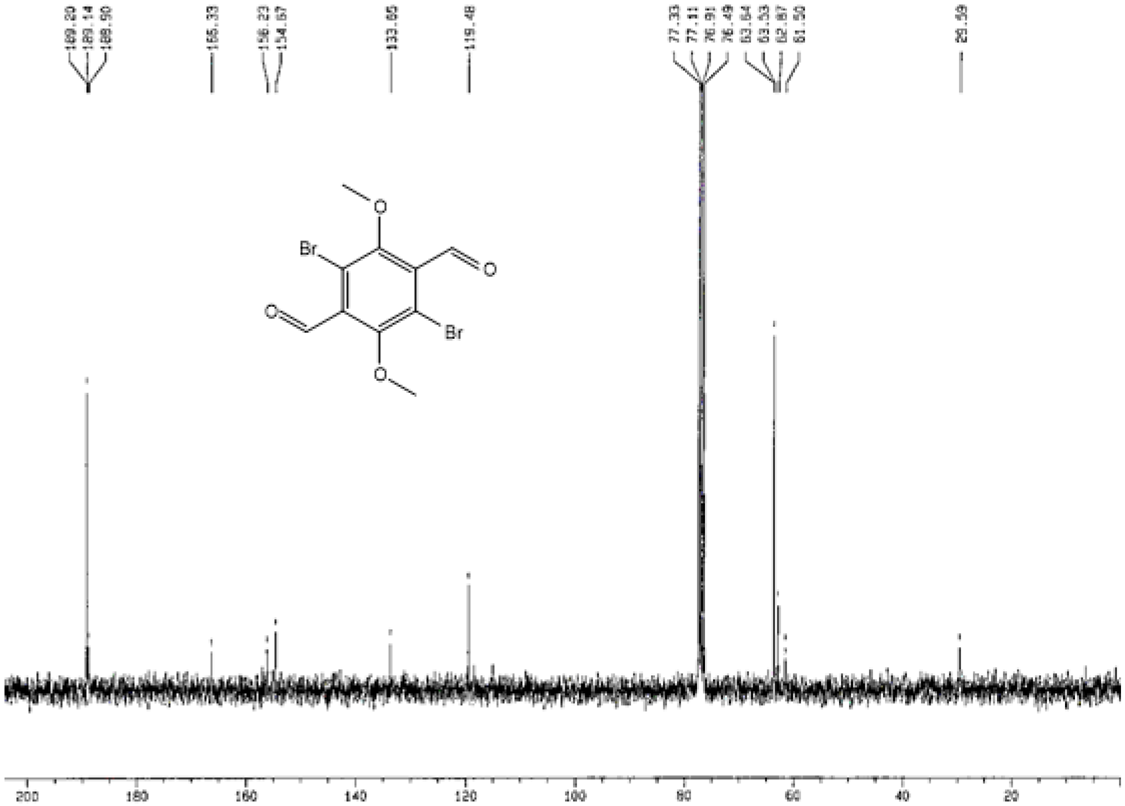

Supplement: Supplementary file 1 [file materials-16-01678-s001.zip › materials-2197043-SI.pdf]
